# Estimating software reliability using size-biased modelling

**DOI:** 10.1080/02664763.2024.2352751

**Published:** 2024-05-10

**Authors:** Soumen Dey, Ashis Kumar Chakraborty

**Affiliations:** a Norwegian University of Life Sciences, s, Norway; b Indian Statistical Institute, Kolkata, India

**Keywords:** Software reliability, software testing, stopping phase, size-biased, Bayesian analysis, bug size

## Abstract

Software testing is an important step in software development where inputs are administered repeatedly to detect bugs present in the software. In this paper, we have considered the estimation of total number of bugs and software reliability as a size-biased sampling problem by introducing the concept of eventual bug size as a latent variable. We have developed a Bayesian generalised linear mixed model (GLMM) using software testing detection data to estimate software reliability and stopping phase. The model uses size-biased approach where the probability of detecting a bug is an increasing function of eventual size of the bug which is as an index for the potential number of inputs that may eventually pass through the bug. We have tested the sensitivity of the reliability estimates by varying the number of inputs and detection probability via a simulation study and have found that the key parameters could be accurately estimated. Further, we have applied our model to two empirical data sets – one from a commercial software and the other from ISRO launch mission software testing data set. The hierarchical modelling approach provides a unified modelling framework that may find applications in other fields (e.g. hydrocarbon explorations) apart from software management.

## 1. Introduction

Software is the fuel of the current world. Economy, technology, transport, communication, medical treatment – all of these essential components of our daily lives are critically dependent on successful execution of software. Most of the modern day devices may not function properly if the concerned software carry bugs. Thus it is not surprising that the estimation of software reliability remains a cornerstone in the field of software development and testing [14,17].

The determination of optimum time for software release remains an interesting field of research [4]. Time between failures (TBF) data have become difficult to collect as the complexity in software development and testing has increased rapidly in past decade. Often the logged information during software testing is test-case specific and consequently discrete in nature [12]. The estimation of optimum duration of software testing under such a discrete set up has also received considerable attention [2,3,6,8]. Recently, [4] proposed a novel optimum testing strategies based on the remaining number of bugs present in the software [4,9]. If a bug remains present in certain path of a software which will rarely be traversed by any inputs to be used by the users, the chances of encountering the bug would also be low. Though, a dependence between bug attribute and its detectability seems plausible and close to reality, it has not been systematically studied yet in the literature.

To account for the probability of a bug to go undetected during software testing, we introduce the notion of ‘eventual size of a bug’ as a latent variable [2]. The eventual size of a bug is defined as the number of inputs that may eventually pass through the bug (or its location) during the entire lifetime of a software, irrespective of whether the bug is detected or not during the testing phase. In other words, if a bug is detected and is subsequently fixed, the eventual size of a bug also includes the inputs that pass through the location where the bug existed. Occasionally, the eventual size of a bug is also referred to as simply ‘the size of the bug’. A software can be considered as a collection of several paths and each input to the software is expected to follow a particular path. In particular, if a particular input is used several times, it can only check whether any bug is present on that particular path or not, and will not be able to check the presence of bugs in other paths as the given input will not traverse in those paths. A newly developed software would require different inputs during software testing to check the existence of bugs in its different paths. Without loss of generality, we may assume that an input can only identify at most one bug which lies on the path that the input would traverse. The size-biased concept was first introduced by [2] in software reliability, although the concept had also been applied in a few other fields of investigation [13].

It is quite natural that a path in a software branches out to several sub-paths at subsequent levels. Consequently, the size of a bug in a path near source is expected to be higher than that of a bug in a sub-path at a deeper level. Thus the size of a bug can also be thought of as an index of how likely a bug could be encountered. Larger the size of the bug, larger should be the probability of detecting the bug, as has been indicated in [3] where a similar approach was applied for discovering fields with rich hydrocarbon contents in the field of producing oil and natural gas.

In a discrete software testing framework, when an input is being administered, it results in either a success (i.e. finding an error) or a failure (i.e. not finding an error). Often, testing of software is carried out into multiple phases, where a series of inputs are tested in each phase and results of each testing are recorded as either a success or a failure [8]. After detecting the bugs, they are debugged at the end of testing within a phase. This process of debugging is known as periodic debugging or interval debugging [5]. Thus the detection of a bug during software testing can be thought of as a outcome of Bernoulli trial (i.e. administering an input), where the probability of a bug being detected is an increasing function of the size of the bug. This is analogous to the size-biased modelling by [13], for modelling identification of species.

A bug on a path that is rarely traversed by an input is likely to be innocuous as far as running of the software is concerned. Thus the reliability of the software does not only depend on the number of bugs remaining in the software, it also depends on the positioning of the bugs ( and consequently on the bug size) [11]. Hence to develop a model for estimating software reliability, both the number of undetected bugs and their total size would be our parameters of interest.

### 1.1. Motivation

The present work was motivated by one key idea that the optimum time to stop software testing and the optimum time to stop drilling in hydrocarbon exploration were found to be analogous [2,3]. It is quite logical to understand that bigger field, in terms of the amount of oil and natural gas that can be obtained after drilling in, are expected to be drilled much ahead compared to the others. Thus size (in terms of the value of the oil and natural gas in the field) plays an important role in identifying the chronological order of the drilling areas. Ideally, this strategy would minimize the overall drilling cost. Similarly, once the size of a bug is appropriately defined (as has been done in earlier paragraphs), the size-biased nature of the problem can be used to model the detection probability of a bug in software testing data. However, the eventual size of a bug remains unknown, which becomes the major challenge to the present problem.

This article is organized as follows. In Section 2, we developed a Bayesian generalized linear mixed model. In Section 3, we provided a description of model fitting and model performance measures used for this study. In Section 4, we showcased the results from a simulation study to assess the performance of our model. We assessed the performance of the models using relative bias, coefficient of variation, and coverage probability. The application of this model was carried out on two empirical software testing data sets. Section 5 illustrates an application of the developed Bayesian model to a commercial software testing data set, and in Section 6, we showed an application of the model to a software testing data set used for Indian space mission software. This article ends with a discussion and conclusion in Section 7.

## 2. Methods

We utilized the hierarchical modelling philosophy to formulate a statistical model to address the problem of estimating the total number of bugs in the presence of imperfect detection of the bugs during software testing process. The developed model can also be used to estimate the remaining eventual bug size that is present in the software, as well as the software reliability. We also provided a new method to predict the stopping phase such that the estimated remaining bug size at that phase remains below a preassigned threshold. Later, we extended the model described above to also accommodate the possible groups of bugs who share the same bug size.

### 2.1. Model description

The model has composed of two hierarchical structure: one for the state process that explains the latent dynamic of the bugs within the software, while the other part corresponds to the observation model explaining the probabilistic structure of the observed software testing data.

#### 2.1.1. State process

Consider *N* number of distinct and independent bugs are present in a particular software and size of each bug is denoted by $S_{i}$, i=1,2,…,N. The eventual size of a bug (or in short, size of a bug) is considered as a latent variable in the model and is needed to be estimated. Let S denotes a vector of these latent variables $S_{1},S_{2},\ldots ,S_{N}$ defining the size of the *N* (unknown) bugs under study. For the ease of computation and other technical advantages (described later), we define N∼Binomial(M, ψ), where *M* represents the maximum possible number of bugs present in the software and *ψ* denotes the inclusion probability to indicate the proportion of *M* that represents the real population of bugs.

#### 2.1.2. Observation process

We suppose that $T_{j},\;j=1,2,\ldots ,Q$ inputs are used for each of the *Q* testing phases. We consider the situation where a present bug can get detected in any of the $T_{j}$ inputs at the *j*th phase, j=1,2,…,Q.

Let $y_{\mathrm{ij}}$ represent the binomial detection outcome for a bug *i* over the $T_{j}$ inputs at phase *j*. If $y_{\mathrm{ij}}>0$, this subsequently implies that $y_{\mathrm{il}}=0$, l=1,2,…,(j−1). It should be noted that after a bug gets detected at the *j*th phase, it is eliminated from the pool of bugs during the debugging at the end of phase *j*. For example, in a software testing, if bug 1 gets detected at phase *j* = 4, we would have $y_{11}=y_{12}=y_{13}=0$ and $y_{14}>0$.

We used the data augmentation approach to model the number *N* of bugs in the software by choosing a large integer *M* to bound *N* and introduced a vector of *M* latent binary variables $z=(z_{1},z_{2},\ldots ,z_{M}{)}^{\prime }$ such that $z_{i}=1$ if individual *i* is a member of the population and $z_{i}=0$ otherwise. We assume that each $z_{i}$ is a realisation of a Bernoulli trial with parameter *ψ*, the inclusion probability. Thus the total number of bugs $N=\sum_{i=1}^{M}z_{i}$ follows the Binomial distribution with parameters *M* and *ψ*.

Data augmentation (DA) is a computational device that enables a convenient Bayesian analysis of detection models where number of bugs (*N*) is unknown. In practice, we should assign a sufficiently large value for the data augmentation parameter *M*, relative to the number of bugs that has been detected. From the binomial model on *N*, E(N)=Mψ with ψ∈(0,1). Therefore, estimate of *ψ* will decrease if *M* is chosen with a higher value. Precautions must be taken when setting the data augmentation value *M*. If a very low value is set, then there maybe a possibility of underestimating the total number of bugs *N* by truncating its posterior distribution which will be concentrated near the upper limit of its support. Similarly, if a very high value is set, it may result in high computational time.

From a practical point of view, data augmentation creates a list of pseudo-bugs that are always available for the MCMC algorithm to use if necessary. That is, these pseudo-bugs leave and enter the population depending on the current values of the model parameters [16].

For the software testing, a binomial model, conditional on $z_{i}=1$, is assumed for each observation $y_{\mathrm{ij}}$,

(1) $$y_{\mathrm{ij}}∼\mathrm{Binomial}(T_{j},p_{i}),$$

where $p_{i}$ denotes the detection probability of the *i*th bug in a phase. However, when $z_{i}=0$, $y_{\mathrm{ij}}$ has degenerate distributions at 0 for each j=1,2,…,Q. The detection probability $p_{i}$ is modelled as an increasing function of the bug size $S_{i}$, since the detection probability directly depends on the size of a bug, that is, more the bug size, higher the detectability.

#### 2.1.3. Model for detection probability

From the definition of bug size, $S_{i}$ is higher if placement of *i*th bug is on a common path near the origin and a number of sub-paths follow subsequently. If *r* denotes the probability of bug detection in any one of the inputs that would eventually pass through the *i*th bug, then the probability of detecting *i*th bug with one input is

(2) $$p_{i}=p(r,S_{i})=1-(1-r{)}^{S_{i}}.$$

The parameter *r* plays the role of a shared parameter across all the bugs and critical for the dependence structure of the nodes in our joint probability model. In addition, the above formulation of $p_{i}$ comes naturally from our definition of bug size and accounts for individual-level heterogeneity in detection probability of the bugs [13]. Note that, here $p_{i}$ is modelled as a monotonically increasing function of $S_{i}$ and when $S_{i}=0$, we have $p_{i}=0$.

We assume that *n* bugs get detected over the *Q* testing phases which is expected to be less than the total number of bugs *N* due to imperfect detection during testing. Consequently, as part of the data augmentation approach, the detection data set $((y_{\mathrm{ij}}){)}_{1\leq i\leq n,1\leq j\leq Q}$ is supplemented with a large number of ‘all-zero’ encounter histories $Y_{\mathrm{rem}}$, an array of ‘all-zero’ detection histories with dimensions (M−n)×Q. We label the zero augmented complete detection data set as Y and the joint density of $Y=((y_{\mathrm{ij}}){)}_{1\leq i\leq M,1\leq j\leq Q}$ is the following:

(3) f(Y | S,z,r)=∏i=1M∏j=1Qf(yij | r, zi, Si)=∏i=1M∏j=1Q{(Tjyij) piyijk (1−pi)Tj−yijk}zi.

#### 2.1.4. Prior density

Bug sizes ( $S_{i}$'s) are usually latent and unobservable. We assign a Poisson–Gamma mixture prior for $S_{i}$ to capture the required level of variability in the latent variable. Consequently, each $S_{i}$ is assumed to follow Poisson distribution with mean $\lambda_{i}$, where each $\lambda_{i}$ is a random draw from Gamma distribution with shape parameter *a* and rate *b* to retain conjugacy. We complete the prior specification by choosing hyperparameter values that lead to highly vague priors, *a* = 0.5 and *b* = 0.01. The prior distributions assigned to the other parameters (e.g. the detection probability *r* and the inclusion probability *ψ*) are assumed to be mutually independent. In particular, we assume a Uniform (0,1) prior for both *r* and *ψ*. We use uniform distributions to specify that none of the values between 0 and 1 can be regarded more likely than others [p. 149][1]. These proper prior specifications ensured propriety of the posteriors.

#### 2.1.5. Posterior density

We recall that each $z_{i}$ follows a Bernoulli distribution with parameter *ψ* independently, i=1,2,…,M and $\pi (z|\psi )=\prod_{i=1}^{M}\pi (z_{i}|\psi )$. The posterior density of parameters {S,Λ,z,r,ψ} can be presented as follows:

(4) π(S,Λ,z,r,ψ∣Y)∝f(Y∣S,z,r) π(S∣Λ,z) π(Λ∣z) π(z∣ψ) π(ψ) π(r)∝∏i=1M[∏j=1Q{(Tjyij) piyij (1−pi)Tj−yij}zi π(Si∣λi,zi) π(λi∣zi) π(zi∣ψ)] π(ψ) π(r),

where π(ψ) and π(r) denote the independent Uniform (0,1) prior densities for the parameters *ψ*, *r* respectively, and $p_{i}=p(r,S_{i})=1-(1-r{)}^{S_{i}}$ (from (2)). Here the joint prior density of $S=(S_{1},S_{2},\ldots ,S_{M}{)}^{\prime }$ and $\Lambda =(\lambda_{1},\lambda_{2},\ldots ,\lambda_{M}{)}^{\prime }$ is presented as follows:

(5) $$\begin{array}{rl} \pi (S|\Lambda ,z)\;\pi (\Lambda |z) & =\prod_{i=1}^{M}\pi (S_{i}|\lambda_{i},z_{i})\;\pi (\lambda_{i}|z_{i})\\ & =\prod_{i=1}^{M}{\left[\left\{\exp(-\lambda_{i})\;\frac{\lambda_{i}^{S_{i}}}{S_{i}!}\right\}\;\cdot \;\left\{\frac{b^{a}}{\Gamma (a)}\;\lambda_{i}^{a-1}\;\exp(-b\lambda_{i})\right\}\right]}^{z_{i}}. \end{array}$$

#### 2.1.6. Estimating the remaining eventual bug size and the stopping phase

In software testing, certain decisions are critical: for example, when should we stop testing, what should be the criteria to stop software testing process. If after the testing and debugging phases, certain bugs remain in the software, it may cause improper functioning of the software even after the market release. Therefore, a decision to optimize software testing and debugging time is an important part of the development process of software.

The above model is well suited to estimate the number of bugs *N*, the detection probability $p_{i}$'s, and bug size $S_{i}$'s. But to estimate the remaining eventual bug size at a later untested phase, we proceed as follows.

We denote *f* as the model for the detection observations for a bug with number of inputs $T_{j}$, j=1,2,…,J, where *J*>*Q*, and $\tilde{y}$ as future observation or alternative detection outcome that could have been obtained during the testing phase. Since the stopping phase (such that the remaining eventual total size of the bugs is less than a threshold, say, *ϵ*) is unknown to the software tester, we assign a sufficiently large value for *J*, considering the available RAM size of the computing device and computing time. The posterior predictive model for a new detection data ${\tilde{y}}_{i}$ for the *i*th bug is then

(6) $$f({\tilde{y}}_{i}\;|\;Y)=\int f({\tilde{y}}_{i}\;|\;\theta )\pi (\theta \;|\;Y)\;d\theta ,$$

where θ denotes the vector of all the parameters r,S,z,ψ and $f({\tilde{y}}_{i}\;|\;Y)$ is the predictive density for ${\tilde{y}}_{i}$ induced by the posterior distribution π(θ | Y).

In practice, we obtain a single posterior replicate ${\tilde{y}}_{i}^{\left(l\right)}$ by drawing from the model $f({\tilde{y}}_{i}\;|\;\theta^{\left(l\right)})$, where $\left\{\theta^{\left(l\right)}\;:\;l=1,2,\ldots ,L\right\}$ represents a set of MCMC draws from the posterior distribution of parameter θ.

We define a set of deterministic binary variables $u_{\mathrm{ij}}$ which takes the value 1 if *i*th bug is detected on or before *j*th phase and 0 otherwise. Total size of the bugs that are detected up to the *j*th phase is then computed as $A_{j}=\sum_{i=1}^{M}S_{i}z_{i}u_{\mathrm{ij}}$, j=1,2,…,J. Consequently, we also compute the total eventual remaining size of the bugs that are not detected up to the *j*th phase, $B_{j}=\sum_{i=1}^{M}S_{i}z_{i}(1-u_{\mathrm{ij}})$, j=1,2,…,J. We obtain the stopping phase, denoted by *k*, such that $B_{k}<\epsilon$ (where *ϵ* is a preassigned threshold). We compute $B_{j}$ for each replicated data set $\left\{{\tilde{y}}_{i}^{\left(l\right)}\;:\;i=1,2,\ldots ,M\right\}$, l=1,2,…,L, thus enabling us to obtain an MCMC sample for both *k* and $\left\{B_{j}\;:\;j=1,2,\ldots ,J\right\}$.

#### 2.1.7. Software reliability

For software testing detection data set, we define software reliability, at a testing phase *j*, as the posterior probability that the total eventual remaining size of the bugs $B_{j}$ (that are not detected up to the *j*th phase) is less than or equal to the prefixed small quantity *ϵ* given the observed detection data Y,

(7) $$\gamma_{j}(\epsilon )=P(B_{j}\leq \epsilon \;|\;Y).$$

Consequently, reliability is a non-decreasing function of threshold *ϵ* and testing phase *j*. Asymptotically, for a fixed *j*, (i) as ϵ→0, $\gamma_{j}(\epsilon )\to 0$ and (ii) as ϵ→∞, $\gamma_{j}(\epsilon )\to 1$. Similarly, for a fixed *ϵ*, if we conduct a very large number of testing phases (i.e. *j* becomes large), reliability $\gamma_{j}(\epsilon )$ will be very close to 1. Of course, this rate of convergence will also depend on the number of testing inputs $T_{j}$ in each phase.

In practice, we compute the reliability from the proportion of iterations where the remaining bug size lies below a threshold *ϵ* using the MCMC samples of the parameters. We use posterior predictive simulation of the detection data $\tilde{y}$ to obtain a series of reliability estimates in future phases (i.e. phases to be conducted after the initial *Q* testing phases), so that one can stop the testing after achieving a desired level of reliability (consequently obtain an estimate of the ‘stopping phase’). We have investigated the sensitivity of the reliability estimates by using different thresholds with a varying number of test inputs.

### 2.2. Modelling for grouped bugs

Often we come across situations where a few bugs are collocated on the same path or same part of the software in such a way that we can assume without loss of generality that each of them have the same bug size. For computational and notational simplicity, we make a transformation of the data set $\left((y_{\mathrm{ij}})\right)$ to $\left(y_{g}^{*}\right)$ where the observed data $y_{g}^{*}$ represents the number of bugs from the *g*th group that are detected. Consequently, we have $y_{g}^{*}∼\mathrm{Binomial}(T_{j(g)},p_{g}^{*})$, $p_{g}^{*}$ denotes the probability of detecting a bug belonging to the *g*th bug group with a single test case and j(g) denotes the corresponding phase to the *g*th group.

Here, we consider a number of distinct group of bugs $N_{G}$ that are present in a software and each bug in a group (say, *g* th) has size $S_{g}^{*}$. Each group of bugs comprises at least one bug. Following Section 2.1.1, we define $N_{G}∼\mathrm{Binomial}(M_{G},\psi )$, where $M_{G}$ is a large positive integer that gives an upper bound to $N_{G}$. The link between $p_{g}$ and the size $S_{g}^{*}$ remains the same as in Section 2.1.3, $p_{g}^{*}=1-(1-r^{*}{)}^{S_{g}^{*}}$. We used the data augmentation approach to model the number of bug groups $N_{G}$ (discussed in Section 2.1.2). The total number of bugs $N^{*}$ has the following expression:

(8) $$N^{*}=n+\sum_{g=1}^{M_{G}}a_{g}z_{g},$$

where *n* denotes the number of bugs detected during the testing period and $a_{g}$ denotes the number of bugs in the *g*th group that went undetected. We utilized the posterior predictive distribution of new detection data ${\tilde{y}}_{g}^{*}$ with density $f({\tilde{y}}_{g}^{*}\;|\;Y^{*})$ to estimate $a_{g}$.

To compute the remaining eventual size, we introduce binary variables $\left((u_{\mathrm{gQ}})\right)$, $g=1,2,\ldots ,M_{G}$, where $u_{\mathrm{gQ}}$ takes the value 1 if *g*th bug group is detected on or before *Q*th phase and takes 0 otherwise. The remaining eventual size is calculated as $B_{Q}=\sum_{g=1}^{M_{G}}S_{g}z_{g}d_{g}(1-u_{\mathrm{gQ}})$, where $d_{g}$ denotes the number of bugs in *g*th bug group.

## 3. Model fitting

The joint posterior density π(S,Λ,z,r,ψ∣Y) in (4) is not in a tractable form. We fitted models using Markov chain Monte Carlo (MCMC) simulations. In particular, we used Gibbs sampling for simulating the parameters from the posterior distribution. The full conditional posterior densities are given in Section 3.1. We used adaptive slice sampler for $S_{i}$'s and random walk Metropolis–Hastings sampler for the other parameters (e.g. *r*). We implemented MCMC computations using NIMBLE [7] in R software [15]. We ran three chains of 10,000 iterations including an initial burn-in phase of 5000 iterations. MCMC convergence and mixing of each model parameters were monitored using the Gelman–Rubin convergence diagnostics $\hat{R}$ [with upper threshold 1.1][10] and MCMC traceplots.

### 3.1. Full conditional posterior densities

The full conditional posterior densities of different parameters are derived below.

- A uniform prior is assumed for *r* over (0, 1). The full conditional of *r* is obtained from (4) and is of a non-standard form
π(r | Y,S,Λ,z,ψ)∝π(r) ∏i=1M∏j=1Q {(Tjyij) piyij (1−pi)Tj−yij}zi∝π(r∗) ∏i=1M∏j=1Q {1−(1−r)Si}ziyij (1−r)ziSi(Tj−yij).
- The $z_{i}$'s are assumed to have independent Bernoulli prior with parameter *ψ*. For all the detected bugs, i.e. *i*'s such that $y_{i0}=\sum_{j=1}^{Q}y_{\mathrm{ij}}>0$, the full conditional of $z_{i}$ has a degenerate distribution at the value 1, denoted by
$$\pi (z_{i}\;|\;y_{i\cdot },y_{i\cdot }>0,S,\Lambda ,z_{\mathrm{rest}},r^{*})=I(z_{i}=1).$$
  For the undetected bugs with all zero detection histories, the full conditional for each $z_{i}$ can be obtained from the expression (4), which yields
$$\begin{array}{rl} & \pi (z_{i}\;|\;y_{i\cdot }=0,S,\Lambda ,z_{\mathrm{rest}},r^{*})\\ & \;\propto {\left[\psi {\left\{1-{\left(\frac{1}{1+\exp(r^{*})}\right)}^{S_{i}}\right\}}^{y_{i\cdot }}{\left(\frac{1}{1+\exp(r^{*})}\right)}^{S_{i}(\sum_{j=1}^{Q}T_{j}-y_{i\cdot })}\right]}^{z_{i}}\\ & \;\times (1-\psi {)}^{1-z_{i}}\\ & \;=\psi_{0i}^{z_{i}}\;(1-\psi_{0i}{)}^{1-z_{i}}, \end{array}$$
  where
$$\psi_{0i}=\frac{\psi {\left\{1-{\left(\frac{1}{1+\exp(r^{*})}\right)}^{S_{i}}\right\}}^{y_{i\cdot }}{\left(\frac{1}{1+\exp(r^{*})}\right)}^{S_{i}(\sum_{j=1}^{Q}T_{j}-y_{i\cdot })}}{\psi {\left\{1-{\left(\frac{1}{1+\exp(r^{*})}\right)}^{S_{i}}\right\}}^{y_{i\cdot }}{\left(\frac{1}{1+\exp(r^{*})}\right)}^{S_{i}(\sum_{j=1}^{Q}T_{j}-y_{i\cdot })}+(1-\psi )}$$
  and $z_{\mathrm{rest}}=\{z_{j}:j\neq i,j=1,2,\ldots ,M\}$. Hence the full conditional distribution of $z_{i}$ is Bernoulli with parameter $\psi_{0i}$. Note that the full conditional distribution of $z_{i}$ is independent of any other $z_{j}$'s (j≠i).
- We have assumed a Uniform prior distribution for *ψ* over the interval (0, 1). The full conditional of *ψ* is obtained from (4) and has the following form:
$$\pi (\psi |Y,S,\Lambda ,z,r^{*})\propto \psi^{\sum_{i=1}^{M}z_{i}}\;(1-\psi {)}^{M-\sum_{i=1}^{M}z_{i}}.$$
  From the above full conditional density, it is clear that Beta $\left(\sum_{i=1}^{M}z_{i}+1,M-\sum_{i=1}^{M}z_{i}+1\right)$ is the full conditional distribution of *ψ*.
- A Poisson prior distribution is assumed for each $S_{i}$ with parameter $\lambda_{i}$. The full conditional distribution for $S_{i}$ is of a non-standard form with density
$$\begin{array}{rl} & \pi (S_{i}|Y,S_{\mathrm{rest}},\Lambda ,z,r^{*})\\ & \;\propto \pi (S_{i}|\lambda_{i},z_{i})\prod_{j=1}^{J}{\left\{1-{\left(\frac{1}{1+\exp(r^{*})}\right)}^{S_{i}}\right\}}^{z_{i}y_{\mathrm{ij}}}{\left(\frac{1}{1+\exp(r^{*})}\right)}^{z_{i}S_{i}(T_{j}-y_{\mathrm{ij}})}\\ & \;\propto {\left\{\exp(-\lambda_{i})\frac{\lambda_{i}^{S_{i}}}{S_{i}!}\right\}}^{z_{i}}{\left\{1-{\left(\frac{1}{1+\exp(r^{*})}\right)}^{S_{i}}\right\}}^{z_{i}y_{i\cdot }}\\ & \;{\left(\frac{1}{1+\exp(r^{*})}\right)}^{z_{i}S_{i}(\sum_{j=1}^{Q}T_{j}-y_{i\cdot })}, \end{array}$$
  where $S_{\mathrm{rest}}=\{S_{m}:l\neq i,l=1,2,\ldots ,M\}$. Note that the full conditional distribution of $S_{i}$ is independent of any other $S_{j}$'s (j≠i).
- A Gamma prior distribution is assumed for each $\lambda_{i}$ with parameters *a* (shape) and *b* (rate). The full conditional distribution for $\lambda_{i}$ also follows a Gamma density because of conjugacy
$$\begin{array}{rl} & \pi (\lambda_{i}|Y,S,\Lambda_{\mathrm{rest}},z,r^{*})\\ & \;\propto \pi (S_{i}|\lambda_{i},z_{i})\;\pi (\lambda |z_{i})\prod_{j=1}^{J}{\left\{1-{\left(\frac{1}{1+\exp(r^{*})}\right)}^{S_{i}}\right\}}^{z_{i}y_{\mathrm{ij}}}\\ & \;{\left(\frac{1}{1+\exp(r^{*})}\right)}^{z_{i}S_{i}(T_{j}-y_{\mathrm{ij}})}\\ & \;\propto {\left[\left\{\exp(-\lambda_{i})\frac{\lambda_{i}^{S_{i}}}{S_{i}!}\right\}\cdot \left\{\frac{b^{a}}{\Gamma (a)}\;\lambda_{i}^{a-1}\;\exp(-b\lambda_{i})\right\}\right]}^{z_{i}}\\ & \;\propto \exp(-z_{i}(b+1)\lambda_{i})\;\lambda_{i}^{z_{i}(a+S_{i}-1)}, \end{array}$$
  where $\Lambda_{\mathrm{rest}}=\{\lambda_{m}:l\neq i,l=1,2,\ldots ,M\}$. The full conditional distribution of $\lambda_{i}$ follows a Gamma distribution with parameters $z_{i}(a+S_{i}-1)+1$ (shape) and $z_{i}(b+1)$ (rate). Note that, each $\lambda_{i}$ is independent of any other $\lambda_{j}$'s (j≠i).

### 3.2. Model performance measures

We used relative bias, coefficient of variation and coverage probability to evaluate the accuracy of the estimates of key parameters. Suppose $\left\{\theta^{\left(r\right)}\;:\;r=1,2,\ldots ,R\right\}$ denotes a set of MCMC draws from the posterior distribution of a scalar parameter *θ*.

**Relative bias**. Relative bias (RB) is calculated as

(9) $$\hat{\mathrm{RB}}(\theta )=\frac{\hat{\theta }-\theta_{0}}{\theta_{0}},$$

where $\hat{\theta }$ denotes the posterior mean $\frac{1}{R}\sum_{r=1}^{R}\theta^{\left(r\right)}$ and $\theta_{0}$ gives the true value.

**Coefficient of variation**. Precision was measured by the coefficient of variation (CV):

(10) $$\hat{\mathrm{CV}}(\theta )=\frac{\hat{\mathrm{SD}}(\theta )}{\hat{\theta }},$$

where $\hat{\mathrm{SD}}(\theta )=\sqrt{\frac{1}{R}\sum_{r=1}^{R}(\theta^{\left(r\right)}-\hat{\theta }{)}^{2}}$ is the posterior standard deviation of parameter *θ*.

**Coverage probability**. Coverage probability was computed as the proportion of model fits for which the estimated 95% Bayesian credible interval of the estimate (CI) contained the true value of *θ*.

## 4. Simulation study

### 4.1. Description of simulated data and simulation scenarios

**Figure 1.**
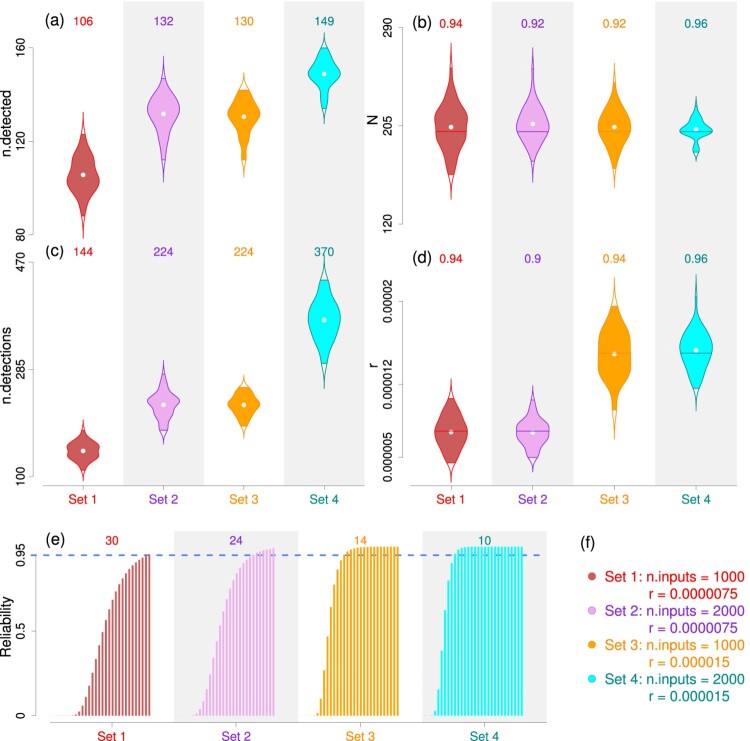
Plots from simulated data analysis. **Panels (a) and (c):** These two panels correspond to violin plots of the number of detected bugs (panel a, ‘n.detected’) and the total number of detections of the bugs, i.e. $\sum_{i=1}^{M}\sum_{j=1}^{J}y_{\mathrm{ij}}$ (panel c, ‘n.detections’) across all the phases of the 50 simulated data sets in each of the four scenarios (Table 1). The mean of the data sets (in panels a and c) are mentioned at the top of each violin. **Panels (b) and (d):** These two panels exhibit violin plots of posterior mean estimates of the total number of bugs *N* (panel b) and detection parameter *r* (panel d) over the 50 model runs to the simulated data sets in each of the four scenarios. The coverage probabilities of the parameters (*N* and *r*) (in panels b and d) are mentioned at the top of each violin. **Panel (e):** The bar plots show the estimates of posterior reliability with threshold ϵ=100 at phases 1, 2, …, 30. The stopping phase (i.e. phase where one can stop software testing) for attaining optimum reliability level 0.95 (indicated by blue dotted line) is mentioned at the top of each bar plot. **Panel (f):** Pre-specified values of the number of inputs (‘n.inputs’) and parameter *r* in each set of simulation scenario that were used to simulate the data sets.

For a complex high-dimensional model such as described in Section 2.1, it would be instrumental to assess model performance with respect to different ranges of the model parameters. We simulated detection data sets of software testing for two values of detection parameter *r*, viz., $0.75\times 10^{-5}$ and $1.5\times 10^{-5}$, and two values of number of inputs in each phase ( $T_{j}$), viz., 1000 and 2000. In total we tested the model with four different simulation scenarios (viz., Sets 1–4) and we simulated a total of 200 data sets (i.e. 50 data sets under each scenario). In each scenario, we assumed a fixed number of bugs *N* = 200 for simulating the detection data of bugs and the software testing was carried out over *Q* = 5 phases. The key details of the simulated data sets are given in Table 1. The number of detected bugs (and also the total number of detections) were higher on average (mean 132) in the set 2 with number of inputs as 2000 as compared to set 1 (mean 106) with number of inputs as 1000, detection parameter *r* remained unchanged in both these two sets at $0.75\times 10^{-5}$. Same phenomenon can be observed for sets 3 (number of inputs = 1000) and 4 (number of inputs = 2000) where $r=1.5\times 10^{-5}$ (see Figure 1 a,c). For estimating the remaining eventual bug size and the stopping phase, the posterior predictive simulations were carried out for 25 additional phases, implying *J* = *Q* + 25 = 30 (see Section 2.1.6).

**Table 1.** Number of detected bugs and the number of total detections (mean, median, 2.5% and 97.5% quantiles) in simulated SCR data sets across 50 repetitions for each simulation scenario.

			No. of detected bugs				No. of detections			
					2.5%	97.5%			2.5%	97.5%
S. no.	No. of inputs	*r*	Mean	Median	Quantile	Quantile	Mean	Median	Quantile	Quantile
1	1000	$0.75\times 10^{-5}$	106	104	92	121	144	145	117	171
2	2000	$0.75\times 10^{-5}$	132	133	114	144	224	228	182	266
3	1000	$1.5\times 10^{-5}$	130	131	113	142	224	224	191	253
4	2000	$1.5\times 10^{-5}$	149	149	135	159	370	370	299	432

### 4.2. Results from simulation study

We fitted our Bayesian size-biased model to each of the 200 simulated data sets using MCMC and *M* is set to 400 for each model fitting. All MCMC samples of the parameters of interest (e.g. the total number of bugs *N*, detection parameter *r*) were obtained after ensuring proper mixing and convergence, with $\hat{R}$ values below 1.1. The posterior estimates of different parameters were obtained using the MCMC chains. The posterior summaries of the total number of bugs *N* and detection parameter *r* for the simulation study are provided in Table 2, respectively and also portrayed in Figure 1.

**Table 2.** Relative bias (mean, median, 2.5% and 97.5% quantiles), coefficient of variation (mean, median, 2.5% and 97.5% quantiles) and coverage probability of the 95% credible interval for the total number of bugs *N* and detection probability parameter *r* across 50 repetitions for each simulation scenario.

			Relative bias				Coefficient of variation				
					2.5%	97.5%			2.5%	97.5%	
S. no.	No. of inputs	*r*	Mean	Median	Quantile	Quantile	Mean	Median	Quantile	Quantile	Coverage probability
Total number of bugs *N*											
1	1000	$0.75\times 10^{-5}$	0.020	0.020	−0.160	0.190	0.100	0.090	0.080	0.120	0.940
2	2000	$0.75\times 10^{-5}$	0.030	0.030	−0.090	0.190	0.060	0.060	0.050	0.070	0.920
3	1000	$1.5\times 10^{-5}$	0.020	0.020	−0.120	0.150	0.060	0.060	0.060	0.070	0.920
4	2000	$1.5\times 10^{-5}$	0.010	0.010	−0.090	0.090	0.050	0.050	0.040	0.050	0.960
Detection probability *r*											
1	1000	$0.75\times 10^{-5}$	−0.010	−0.010	−0.360	0.370	0.190	0.190	0.160	0.240	0.940
2	2000	$0.75\times 10^{-5}$	−0.020	−0.010	−0.320	0.320	0.150	0.150	0.130	0.170	0.900
3	1000	$1.5\times 10^{-5}$	0	−0.010	−0.330	0.270	0.150	0.150	0.130	0.170	0.940
4	2000	$1.5\times 10^{-5}$	0.020	0.020	−0.180	0.220	0.130	0.130	0.110	0.150	0.960

The relative bias and coefficient of variation of *N* and *r* are estimated for each of the 50 replicates in each set. The relative bias estimates of *N* in each set varied between: ( –16%, 19%) in set 1, ( –9%, 19%) in set 2, ( –12%, 15%) in set 3, ( –9%, 9%) in set 4) and the coefficient of variation of *N* in each set varied between: (8%, 12%) in set 1, (5%, 7%) in set 2, (6%, 7%) in set 3, (4%, 5%) in set 4. The relative bias estimates of *r* in each set varied between: ( –36%, 37%) in set 1, ( –32%, 32%) in set 2, ( –33%, 27%) in set 3), ( –18%, 22%) in set 4 and the coefficient of variation of *r* in each set varied between: (16%, 24%) in set 1, (13%, 17%) in set 2, (13%, 17%) in set 3, (11%, 15%) in set 4. In Figure 1, the posterior mean estimates of *N* and *r* over 50 simulations were plotted as violins to show their distributions. It is evident from these plots that as the number of inputs and detection probability increases, the estimates of *N* and *r* get more accurate. Coverage probabilities of both *N* and *r* were higher than 90% in each of the scenarios (these are mentioned at the top of each violins in Figure 1).

We estimated the reliability at the end of each phase and also at different possible future phases (assuming a pre-specified number of test cases in each phases). It is important to mention that the estimation of reliability heavily depends on the pre-specified threshold and the number of test cases used during the future phases (that would be conducted after the first five phases already conducted). Here we have assumed that the number of test cases in each future phase to be the same as the number of inputs in the respective scenario.

The reliability (i.e. posterior probability of the remaining size lying below a threshold) is a non-decreasing function of testing phase index, since remaining bug size gets reduced with more bugs being detected in subsequent testing phases. We found the reliability estimates to attain the targeted 95% level (with threshold 100) to be varying with respect to different simulation scenarios (Figure 1). For instance, the reliability estimate attained the optimum 95% level (with threshold 100) at phase 30 in set 1, implying the developer would need to continue software testing for 25 more future phases (after the 5 testing phases already conducted) to attain optimum software reliability level. Hence the stopping phase was estimated as 30. For other sets, the estimates of the stopping phases were at phase 24 (set 2), phase 14 (set 3) and phase 10 (set 4).

## 5. Application to commercial software testing empirical data

### 5.1. Data description

The commercial software testing data set consisted a total of 8757 test inputs detailed with build number, case id, severity, cycle, result of test, defect id, etc. In this data, the severity of a path is broadly divided into three categories, namely, simple, medium and complex depending on the effect of the bug if it is not debugged before marketing the software. The software testing process had four cycles namely Cycle 1, Cycle 2, Cycle 3 and Cycle 4 – the terminology is equivalent to the different phases of testing described in Section 2. After each cycle, the bugs that were identified during the cycle are debugged as mentioned in Section 2. In total, 226 bugs were detected with 737 detections among them.

### 5.2. Results from commercial software testing data analysis

**Figure 2.**
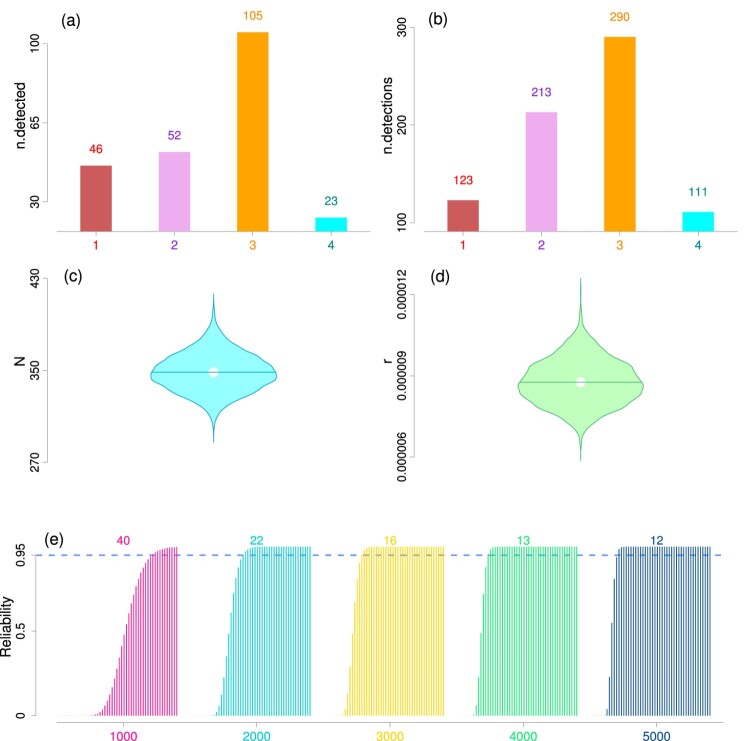
Plots from commercial software testing data analysis. **Panels (a) and (b):** The bar plots of the number of detected bugs (panel a, ‘n.detected’) and the total number of detections of the bugs, i.e. $\sum_{i=1}^{M}\sum_{j=1}^{J}y_{\mathrm{ij}}$ (panel b, ‘n.detections’) across all the testing phases of the commercial software testing data set. **Panels (c) and (d):** These two panels exhibit the violin plots of posterior MCMC samples of the total number of bugs *N* (panel c) and detection parameter *r* (panel d). **Panel (e):** The bar plots show the estimates of posterior reliability with threshold ϵ=100 at phases 1, 2, …, 50. The stopping phase (i.e. phase where one can stop software testing) for attaining optimum reliability level 0.95 (indicated by blue dotted line) is mentioned at the top of each bar plot. Each bar plot corresponds to a distinct number of test inputs that were used in the future phases. In particular, the different inputs used for creating the plots are 1000, 2000, …, 5000 and these are also mentioned along the *x*-axis.

The posterior estimates of the main parameters *N*, *ψ*, *r* and $B_{4}$ are provided in Table 3 and visually portrayed in Figure 2. The posterior mean estimate of the total number of bugs was 348 with a 95% credible interval (317, 382) and its posterior density was plotted as violin in Figure 2(c). The posterior mean of inclusion probability *ψ* was estimated at 0.696 with a 95% credible interval (0.618, 0.774). The estimate of *ψ* also confirmed that the prefixed upper bound *M* = 500 was sufficiently large enough to not to influence in the estimation of *N*. Although the posterior mean estimate of size-biased detection model parameter *r* was estimated at a very small magnitude $8.761\times 10^{-6}$ (95% CI: ( $7.261\times 10^{-6}$, $10.439\times 10^{-6}$)), we had coded the parameter with a logistic transformation to retain the accuracy in estimation and MCMC mixing. We displayed the posterior density of *r* with violin plots in Figure 2(d). Both the violins showed moderately symmetric pattern in the posterior distribution of these two key parameters *N* and *r*. The remaining eventual bug size after the four testing phases was estimated as 703 with a 95% credible interval (457, 1006). Here we have assumed that the number of test cases in each future phase to be 3000 to resemble with the observed data set.

**Table 3.** Estimates of different parameters in the data analysis of commercial software testing data (Section 5).

Parameter	Mean	SD	2.5%	97.5%
			Quantile	Quantile
*N*	348	17	317	382
*ψ*	0.696	0.040	0.618	0.774
*r*	$8.761\times 10^{-6}$	$8.331\times 10^{-7}$	$7.261\times 10^{-6}$	$1.044\times 10^{-5}$
$B_{4}$	703	141	457	1006

The bar plots in Figure 2(e) show the posterior reliability estimates with threshold ϵ=100 at phases 1, 2, …, 50. We found the reliability to attain the target 95% level at phase 16 if we would have continued with 3000 test cases in each phase, implying the developer would need to continue software testing for 12 more future phases (after the 4 testing phases already conducted) to attain the targeted software reliability level. Hence, the stopping phase was estimated as 16. The reliability took much longer (40 phases) to reach the targeted 95% level (optimum reliability level, showed in blue dotted line in the Figure 2e) with 1000 test cases in each phase, and took only 12 phases with 5000 test cases in each phase (these results are provided in the appendix). This also revealed that it takes approximately 36,000 future test cases to attain the targeted reliability of 95%.

## 6. Application to ISRO mission empirical data

### 6.1. Data description

**Figure 3.**
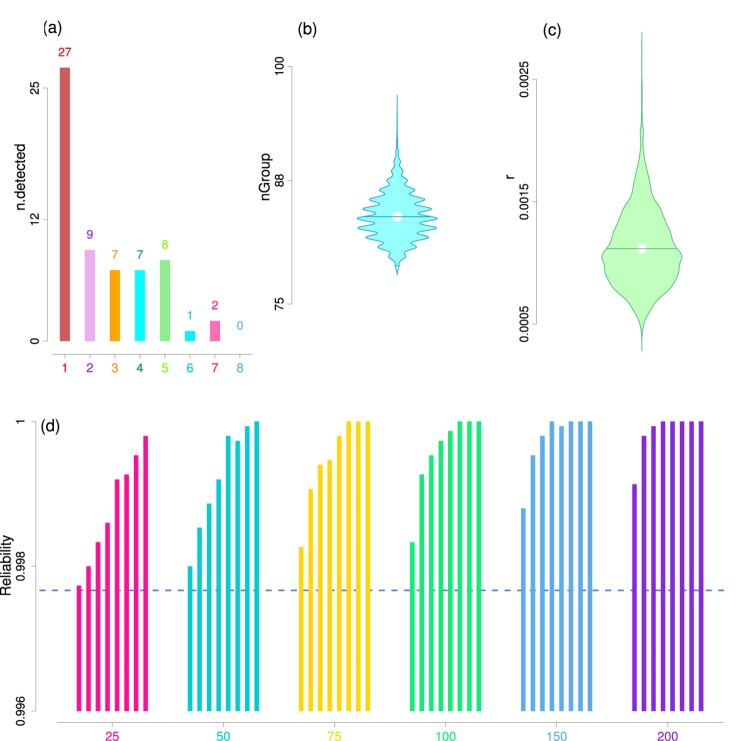
Plots from ISRO mission software testing data analysis. **Panel (a):** The bar plot of the number of detected bugs in each testing phase of the ISRO mission data set. **Panels (b) and (c):** These two panels exhibit the violin plots of posterior MCMC samples of the total number of bug groups ‘nGroup’ (panel b) and detection parameter *r* (panel c). **Panel (d):** The six bar plots show the estimates of posterior reliability with different thresholds 25, 50, 75, 100, 150, 200 respectively. The horizontal dotted line represents the reliability estimate 0.998 after first eight testing phases. The eight bars in each bar plot correspond to different numbers of future test cases 25, 50, 75, 100, 150, 200, 250, 300 respectively.

The ISRO mission software testing data set consisted of the outcomes from software testing conducted on each of the five software during 35 missions. Each of the five software had been updated and tested before executions of the different launch missions. There were three primary stages of software testing: (i) Stage A, where a group of experts manually tested each of these software in search of potential bugs in them, (ii) Stage B, where different parts or modules of these software were tested, (iii) Stage C, where numerous inputs were run through the software in seven different phases, viz., phases 1,2,…,7. Different number of bugs were detected during these three primary stages: $n_{A}=33$ bugs were detected during stage A, $n_{B}=27$ bugs were detected during stage B and $n_{C}=34$ bugs were detected during stage C (where the phase specific segregation is as the following: $n_{C1}=9$, $n_{C2}=7$, $n_{C3}=7$, $n_{C4}=8$, $n_{C5}=1$, $n_{C6}=2$, $n_{C7}=0$). The bar plot in Figure 3(a) displays a visual illustration of the number of detected bugs in different phases. For our analysis, we consider the software testing detection data from stage B and seven phases of stage C (i.e. in total *Q* = 8 testing phases) in total as observed data set. We use the detections during stage A as deterministic constant because of the lack of probabilistic structure of this testing phase.

### 6.2. Results from ISRO mission data analysis

We applied the grouped version of our size-biased model (Section 2.2) to ISRO mission data set which was perfectly suited for applying this model. The different missions and software used in those missions and the different phases of testing – each contributed to the variation of sizes between these groups of bugs. In the observed data set, we considered the detections corresponding to the triplet (mission, software, testing phase) as a ‘group’ of bugs. Each bug in a single group was assumed to have a constant eventual size. Any change in either mission, software or phase was considered as a different group. As it was not possible to extend the number of phases indefinitely by construction of the ISRO mission software testing, we obtained the number of future test inputs required to attain 95% reliability level. These test cases would need to be administered before a future launch mission or after a software update.

The posterior mean of number of groups of bugs was estimated at 84 with a 95% credible interval (80, 89) (see Table 4 and Figure 3 b). The posterior mean estimate of *ψ* was 0.257 with a 95% credible interval (0.195, 0.323). This also confirmed the pre-specified upper bound $M_{G}=200$ for the number of groups to be appropriate. The size-biased detection model parameter was estimated as $1.102\times 10^{-3}$ with a 95% credible interval ( $6.439\times 10^{-4}$, $1.807\times 10^{-3}$) (Figure 3 c). The total number of bugs present was estimated as 94 with 95% credible interval (94,95) which is highly precise.

**Table 4.** Estimates of different parameters in the ISRO mission software testing data analysis (Section 6).

Parameter	Mean	SD	2.5%	97.5%
			Quantile	Quantile
$N_{G}$	84	2	80.000	89.000
*ψ*	0.257	0.032	0.195	0.323
*N*	94	1	94.000	95.000
*r*	$1.102\times 10^{-3}$	$3.006\times 10^{-4}$	$6.439\times 10^{-4}$	$1.807\times 10^{-3}$

The reliability of the software was estimated as 0.998 after the eight testing phases (including module testing and seven phases of simulation testing) with threshold ϵ=25. The high magnitude of reliability was to be expected due to the nature of thorough scrutiny and due diligence which the software had to pass through during the rigorous testing of various ISRO launch mission software. We also showed that reliability increases with the increase in number of future test cases (Figure 3 d).

## 7. Discussion

We described a Bayesian generalized linear mixed model that can be applied to software testing detection data set to explicitly model and estimate the total number of bugs, detection probability and latent size of the bugs. The model also allows estimation of software reliability for any given threshold (Section 2.1). Consequently, we could obtain an estimate of the stopping phase providing the number of additional phases of testing are required to achieve an optimum reliability level (say 0.95).

We showed via a simulation study that the parameters of interest (e.g. *N*, *r*, reliability) can be accurately estimated by our model. Number of inputs plays a key role in software testing in general, as higher number of inputs boosts the probability of detecting of bugs (Table 1). This also led to more accurate estimation of the model parameters, which can be observed in the lower magnitude of CV estimates of *N* and *r* with higher number of inputs (Table 2). Further, we also noted that, in such scenarios, threshold reliability level was attained comparatively quicker than the scenarios with lower number of inputs (Figure 1e).

Size biased model fitted to empirical software testing data of bugs yielded satisfactory estimates of the key parameters. However, we noted that the software testing conducted was rather inefficient since the estimated software reliability was approximately near zero after the first four phases of testing (Figure 2). We anticipate that some major bugs (with moderately large size) were still present. We recommend to continue testing for at least 36,000–40,000 more test cases (which could be broken down into multiple phases) to attain the desired software reliability level 95%.

On the contrary, software reliability estimates of ISRO mission software were found to be extremely high (i.e. 0.998) after the first eight testing phases, demonstrating the advantage of efficient software testing. Our finding that the number of bugs detected was almost equal to the true number of bugs available to be detected also supports this.

The developed model can also be used for similar problems in the other fields. For instance, in hydrocarbon exploration, digging a field can be considered analogous with testing a software with different inputs, outcome of which can be considered either as a success (implying sufficient hydrocarbon has been found after digging) or as a failure (implying that the digging did not yield sufficient hydrocarbon which may be viable).

As with most Bayesian analysis, the choice of priors may affect a few of the parameter estimates (for instance, $A_{j}$ and $B_{j}$), although we have used objective priors to the parameters. Here, the choice of threshold depends on the model user. We suggest to conduct a few preliminary runs with varying phases of detection data (i.e. different subsets to the full data) to observe the sensitivity in reliability estimates as the other phases of detection data are sequentially included in the model fitting. We believe these preliminary reliability numbers from different model runs would be helpful for the model user to decide the threshold to stop testing.

Given the enormous amount of interest in software testing in technology sector, our size-biased model could be very useful to provide accurate estimates of the number of present bugs as well as software reliability. Our model used the Bayesian paradigm which added the required flexibility to estimate a large number of model parameters. Although we found the parameter estimates to be moderately robust, we recommend to conduct a prior sensitivity study before the application of the size-biased model.

## Supplementary Material

Supplemental Material

## Data Availability

R codes for generating simulated data and data analysis are provided in the online supplementary material and also can be found in GitHub (https://github.com/soumenstat89/size_biased). The two empirical data sets on software testing can also be accessed from the same GitHub repository.
