# Supplementary material for: Estimating software reliability using size-biased modelling
Source: J Appl Stat. 2024 May 10;51(16):3386–406. doi: 10.1080/02664763.2024.2352751 (PMC11610320; doi:10.1080/02664763.2024.2352751)
Supplement: Supplemental Material [file CJAS_A_2352751_SM6906.pdf]

Appendix

Estimating Software Reliability Using Size-biased Modelling

Soumen Dey *[1] and Ashis Kumar Chakraborty †[2]

[1] Norwegian University of Life Sciences, Ås, Norway

[2] Indian Statistical Institute, Kolkata, India

*E-mail: *soumenstat89@gmail.com* Orcid: https://orcid.org/0000-0001-6270-2356
†Orcid: https://orcid.org/0000-0002-2003-1336

Appendix A1 Flowchart of the hierarchical model

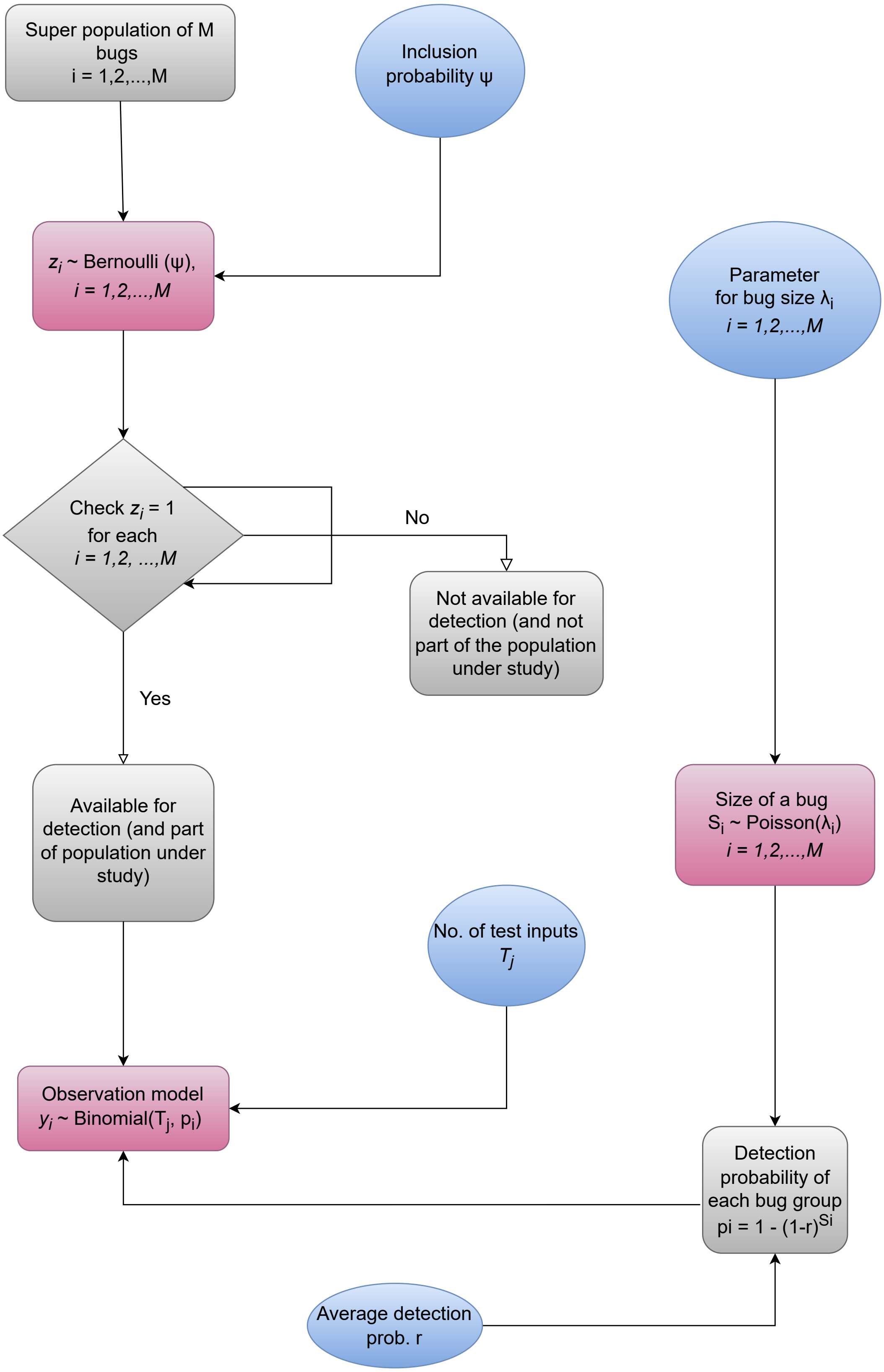

Appendix A2 Example of the computation of remaining bug size in grouped version of the size-biased model

For this example, we extract a subset of the ISRO data set to show the computation of remaining bug size B_j. We consider the total number of phases Q to be 8 (i.e., module testing (MT) and 7 phases of simulation testing (ST)), as it was in the data set. At the start, we set an upper bound for the total number of bug-groups $M = 6$.

Consider the following: One bug got detected in each of missions C12 and C17 on phase 5 (i.e., Stress Oils) of software 2, implying $d_1 = d_2 = 1$. Six other bugs got detected in mission C17 on phase 3 (i.e., SFIT) of software 4, implying $d_3 = 6$. Here number of bugs detected $n = 1 + 1 + 6 = 8$.

Table A1: Example bug data

Bug group	Mission	Software	Phase	No. of bugs detected
$i = 1$	C12	2	5 (Stress Oils)	1
$i = 2$	C17	2	5 (Stress Oils)	1
$i = 3$	C17	4	3 (SFIT)	6

Bug-group $i = 1$ got detected at phase 5, then $u_{1,1} = u_{1,2} = u_{1,3} = u_{1,4} = 0$ and $u_{1,5} = u_{1,6} = u_{1,7} = u_{1,8} = 1$. Bug-group $i = 2$ also got detected at phase 5, then $u_{2,1} = u_{2,2} = u_{2,3} = u_{2,4} = 0$ and $u_{2,5} = u_{2,6} = u_{2,7} = u_{2,8} = 1$. Lastly, bug-group $i = 3$ got detected at phase 3, then $u_{3,1} = u_{3,2} = 0$ and $u_{3,3} = u_{3,4} = u_{3,5} = u_{3,6} = u_{3,7} = u_{3,8} = 1$.

Remaining eventual size B_j for j-th phase can be computed using the following expression

$$B_j = \sum_{i=1}^{M} S_i z_i d_i (1 - u_{ij}),$$

where j can take any value from $1, 2, \ldots, 8$. The sum in the above expression have $M = 6$

terms corresponding to $i = 1, 2, \ldots, M$. Consequently we have,

$$u_{4,1} = u_{4,2} = \cdots = u_{4,8} = 0$$

$$u_{5,1} = u_{5,2} = \cdots = u_{5,8} = 0$$

$$u_{6,1} = u_{6,2} = \cdots = u_{6,8} = 0$$

Since three bug-groups (for $i = 1, 2, 3$) are detected, we have $z_1 = z_2 = z_3 = 1$. The bug-groups corresponding to $i = 4, 5, 6$ are not detected, value of z_i and no. of bugs d_i in those groups would be predicted by the model. Assume, the model predicted $z_4 = 0, z_5 = z_6 = 1$, and $d_4 = 0, d_5 = 2, d_6 = 4$. Bug size S_i of each bug in any bug-group is also unknown and would also be predicted by the model. We assume the estimated value of $S_1 = S_2 = S_3 = S_4 = S_5 = S_6 = 100$. Next we compute the remaining eventual bug size B_j.

For phase $j = 8$,

$$\begin{aligned} B_8 &= S_1 z_1 d_1 (1 - u_{1,8}) + S_2 z_2 d_2 (1 - u_{2,8}) + S_3 z_3 d_3 (1 - u_{3,8}) + S_4 z_4 d_4 (1 - u_{4,8}) \\ &\quad + S_5 z_5 d_5 (1 - u_{5,8}) + S_6 z_6 d_6 (1 - u_{6,8}) \\ &= \{100 \times 1 \times 1 \times (1-1)\} + \{100 \times 1 \times 1 \times (1-1)\} + \{100 \times 1 \times 6 \times (1-1)\} \\ &\quad + \{100 \times 0 \times 0 \times (1-0)\} + \{100 \times 1 \times 2 \times (1-0)\} + \{100 \times 1 \times 4 \times (1-0)\} \\ &= 600. \end{aligned}$$

Although the other B_j's are not needed for our analysis, below are some more calculations for understanding.

For phase $j = 1$,

$$\begin{aligned} B_1 &= S_1 z_1 d_1 (1 - u_{1,1}) + S_2 z_2 d_2 (1 - u_{2,1}) + S_3 z_3 d_3 (1 - u_{3,1}) + S_4 z_4 d_4 (1 - u_{4,1}) \\ &\quad + S_5 z_5 d_5 (1 - u_{5,1}) + S_6 z_6 d_6 (1 - u_{6,1}) \\ &= \{100 \times 1 \times 1 \times (1-0)\} + \{100 \times 1 \times 1 \times (1-0)\} + \{100 \times 1 \times 6 \times (1-0)\} \\ &\quad + \{100 \times 0 \times 0 \times (1-0)\} + \{100 \times 1 \times 2 \times (1-0)\} + \{100 \times 1 \times 4 \times (1-0)\} \\ &= 1400. \end{aligned}$$

For phase $j = 3$,

$$
\begin{aligned}
B_3 &= S_1 z_1 d_1 (1 - u_{1,3}) + S_2 z_2 d_2 (1 - u_{2,3}) + S_3 z_3 d_3 (1 - u_{3,3}) + S_4 z_4 d_4 (1 - u_{4,3}) \\
&\quad + S_5 z_5 d_5 (1 - u_{5,3}) + S_6 z_6 d_6 (1 - u_{6,3}) \\
&= \{100 \times 1 \times 1 \times (1 - 0)\} + \{100 \times 1 \times 1 \times (1 - 0)\} + \{100 \times 1 \times 6 \times (1 - 1)\} \\
&\quad + \{100 \times 0 \times 0 \times (1 - 0)\} + \{100 \times 1 \times 2 \times (1 - 0)\} + \{100 \times 1 \times 4 \times (1 - 0)\} \\
&= 800.
\end{aligned}
$$

Appendix A3 Results

Appendix A3.1 Results from Software testing empirical data analysis

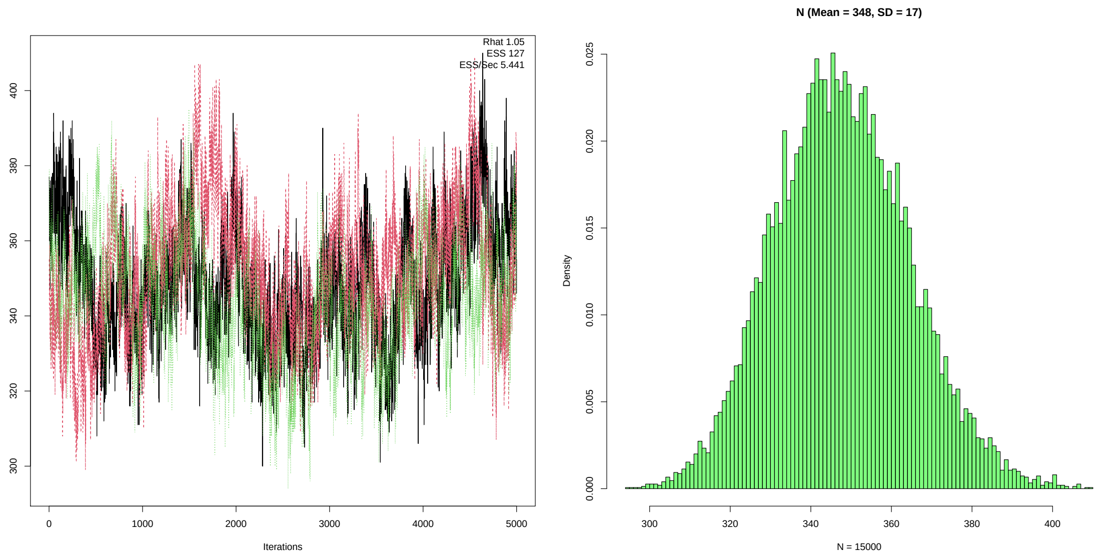

Figure A1: MCMC traceplots and estimated density curve of N.

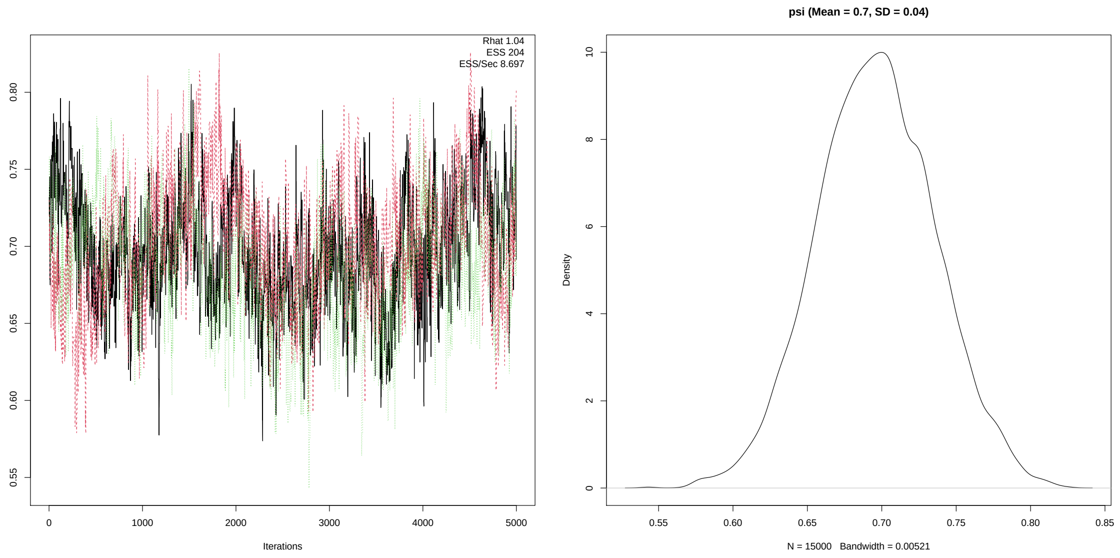

Figure A2: MCMC traceplots and estimated density curve of ψ.

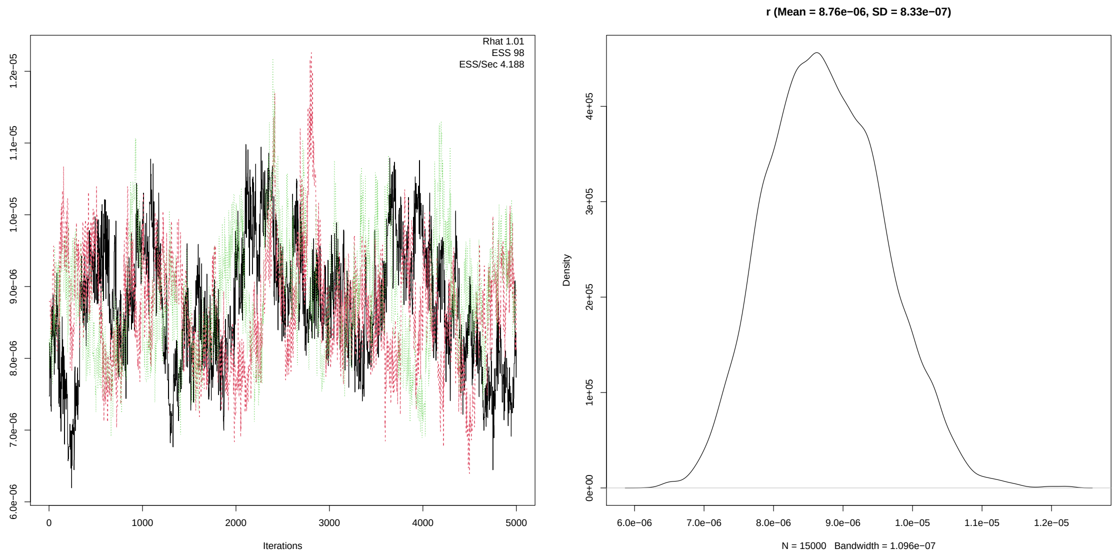

Figure A3: MCMC traceplots and estimated density curve of r.

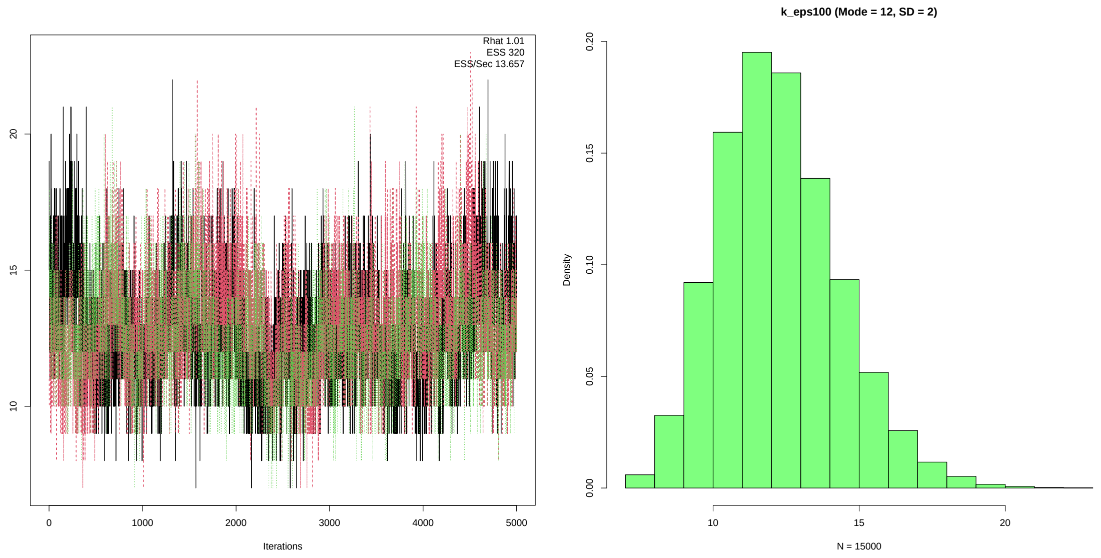

Figure A4: MCMC traceplots and estimated density curve of k with threshold 100 and number of test cases in each future phase = 3000.

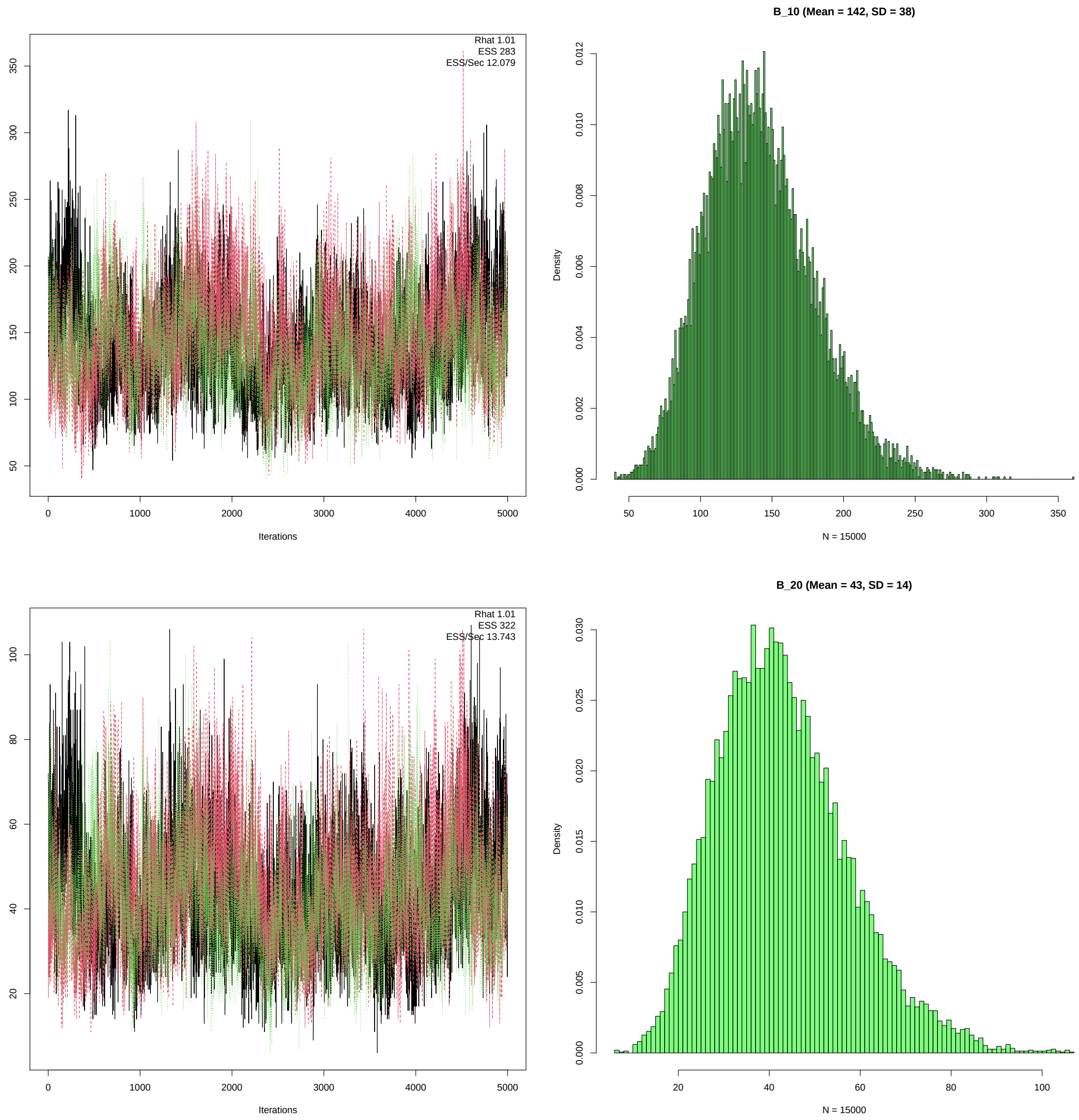

Figure A5: MCMC traceplots and estimated density curve of B_{10} and B_{20}.

Appendix A3.2 Results from ISRO mission empirical data analysis

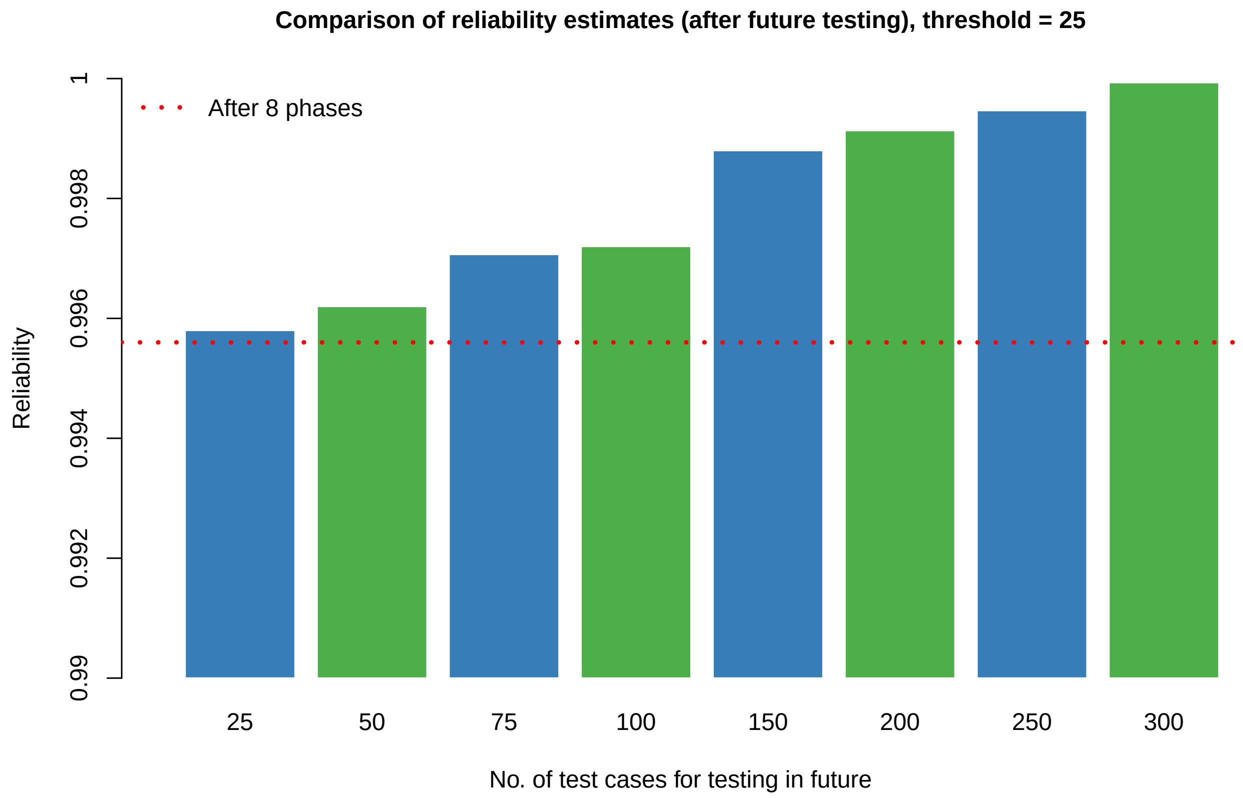

Figure A6: Reliability at threshold = 25. Reliability estimate after 8 phases = 0.995.

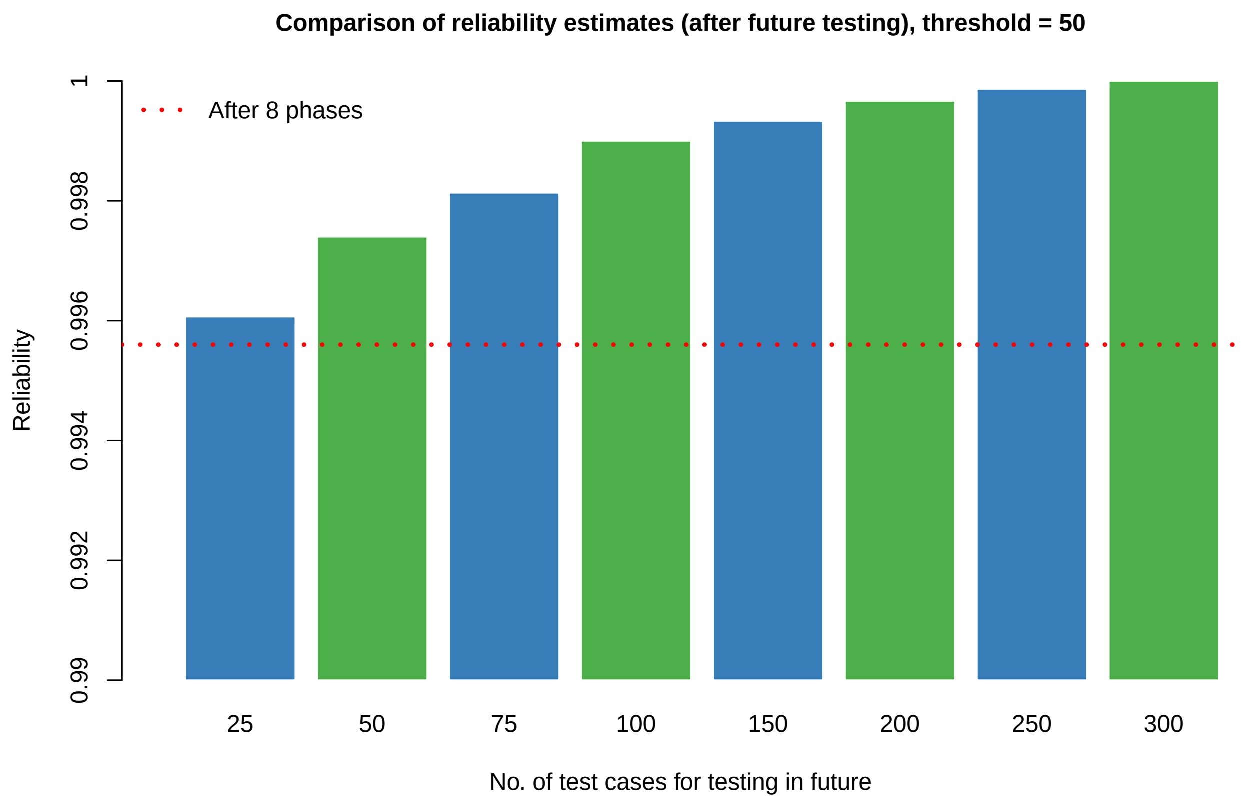

Figure A7: Reliability at threshold = 50. Reliability estimate after 8 phases = 0.995.

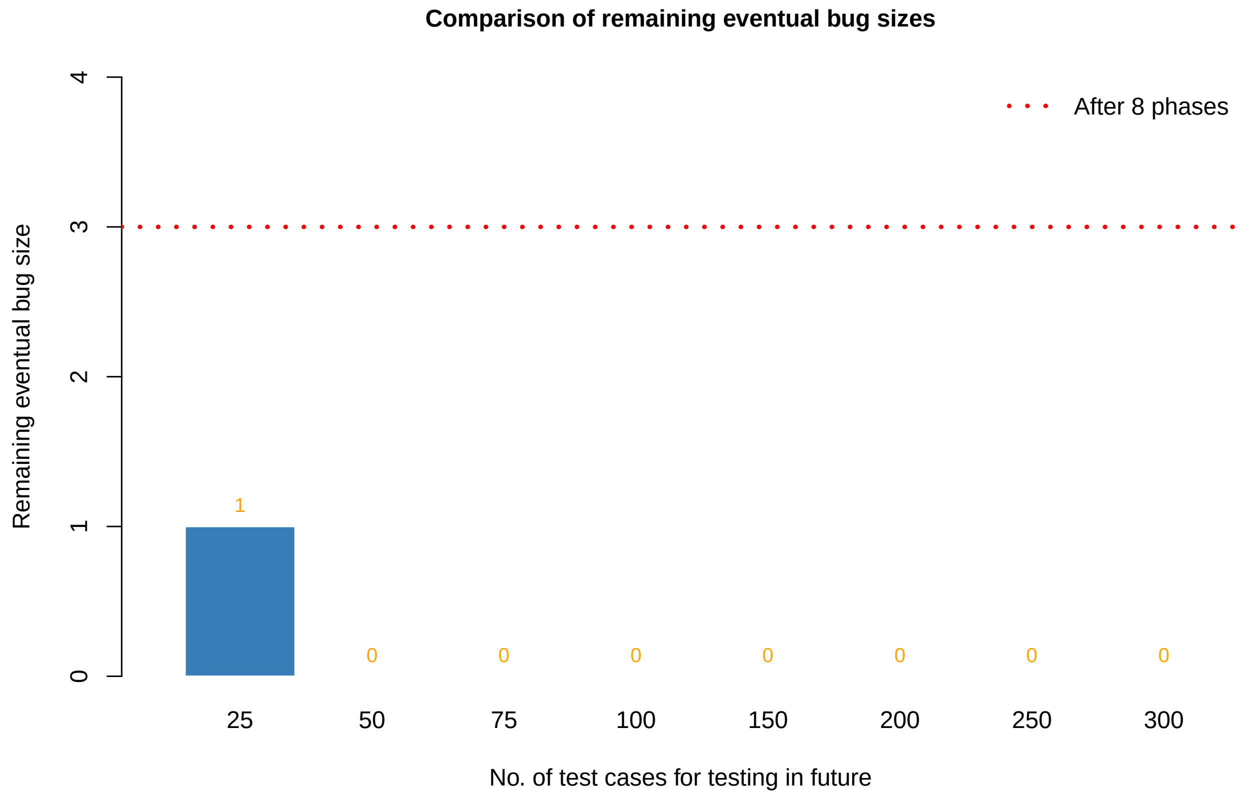

Figure A8: Remaining eventual size (Mean = 3, after 8 phases)

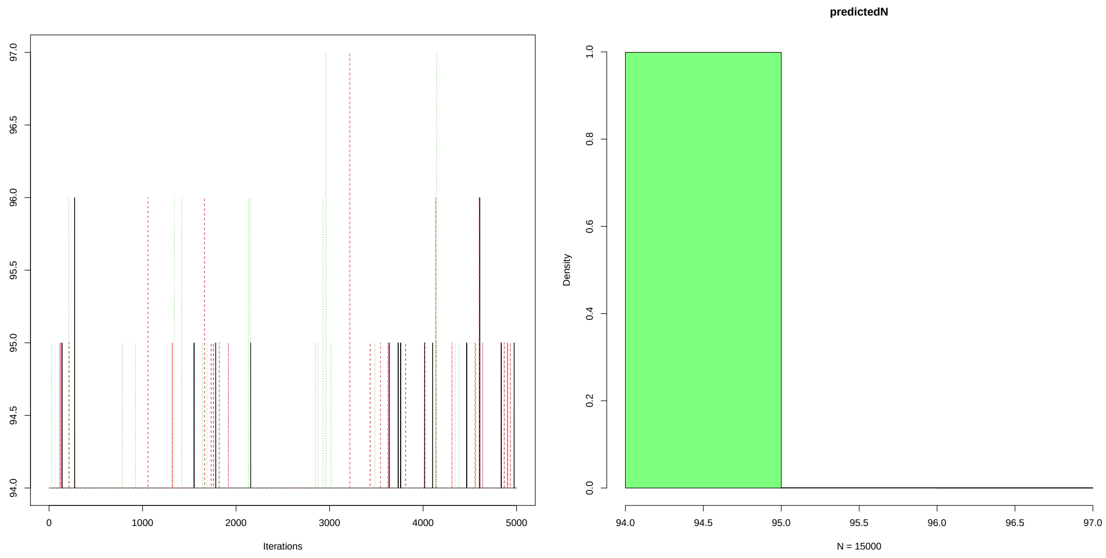

Figure A9: MCMC traceplots and estimated density curve of total number of bugs N (Posterior mean = 94,Rhat = 1.03, ESS = 13613, ESS/Sec = 1764.479.

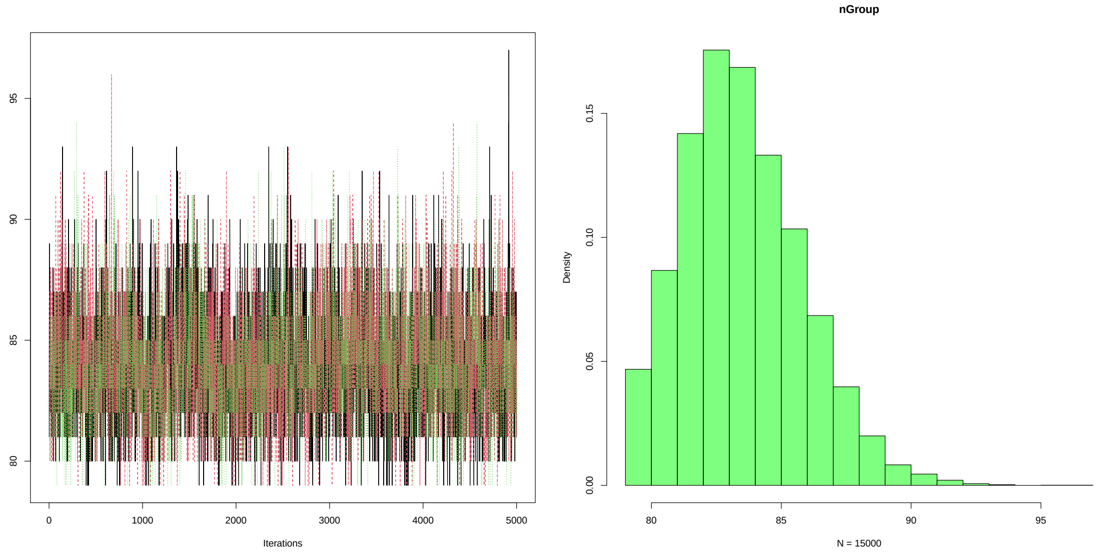

Figure A10: MCMC traceplots and estimated density curve of number of groups of bugs $n.group$ (Posterior mean = 84, Rhat = 1, ESS = 2648, ESS/Sec = 343.215.

References

[1] A.K. Chakraborty, G.K. Basak, and S. Das, "Bayesian optimum stopping rule for software release", DOI no : 10.1007/s12597-018-00353-0,2019.

[2] A.K. Chakraborty, "Software Quality Testing and Remedies", PhD thesis, 1996.

[3] A.K. Chakraborty and T.S. Arthanari, ”Optimum testing time for software under an exploration model”, Opsearch,vol. 31, pp. 202-214, 1994.

[4] S.R. Dalal and C.L. Mallows, ”When should one stop testing software?”, Journal of the American Statistical Association, vol. 83, no. 403, pp. 872-879, 1988.

[5] S. Das, D. Sengupta, and A. Dewanji, ”Optimum release time of a software under periodic debugging schedule”, Communications in Statistics-Simulation and Computation, pp. 1-19, 2017.

[6] S. Das, A. Dewanji, and A.K. Chakraborty, ”Software Reliability Modeling with Periodic Debugging Shedule”. IEEE Transactions On Reliability, vol. 65, no. 3, pp. 1449-1456, 2016.

[7] A. Dewanji, D. Sengupta, and A.K. Chakraborty, ” A discrete time model for software reliability with application to a flight control software”, Applied Stochastic Models in Business and Industry, vol. 27, pp. 723-731, 2011.

[8] B. Littlewood,”Software Reliability Model for modular program structure”, IEEE Transactions on Reliability, vol. 28, no. 3, pp. 241-246, 1979.

[9] T.K. Nayak, ”Estimation of Population Size by recapture sampling”, Biometrika, vol. 75, no. 1, pp. 113-120, 1988.

[10] G.P. Patil and C.R. Rao, ”Weighted Distributions and Size-Biased Sampling with Applications to Wildlife Populations and Human Families”, Biometrics vol. 34, no. 2, pp. 179-189, 1978.

[11] Pham H.(2000), Software reliability, Springer

[12] Yamada Shigeru (2014), Software Reliability Modeling, Springer, Japan.

[13] Eom Heung-seop, Gee-yong Park , Seung-cheol Jang , Han Seong Son, and Hyun Gook Kang (2013), V&V-based remaining fault estimation model for safety–critical software of a nuclear power plant, Annals of Neuclear Energy,51, 38-59.
